# Bacterial Cellulose-Based Nanocomposites Containing Ceria and Their Use in the Process of Stem Cell Proliferation

**DOI:** 10.3390/polym13121999

**Published:** 2021-06-18

**Authors:** Iosif V. Gofman, Alexandra L. Nikolaeva, Albert K. Khripunov, Elena M. Ivan’kova, Anton S. Shabunin, Alexander V. Yakimansky, Dmitriy P. Romanov, Anton L. Popov, Artem M. Ermakov, Sergey O. Solomevich, Pavel M. Bychkovsky, Alexander E. Baranchikov, Vladimir K. Ivanov

**Affiliations:** 1 Institute of Macromolecular Compounds, Russian Academy of Sciences, 199004 Saint Petersburg, Russia; a.l.nikolaeva@imc.macro.ru (A.L.N.); Khripunov@hq.macro.ru (A.K.K.); ivelen@mail.ru (E.M.I.); yakimansky@yahoo.com (A.V.Y.); 2 H. Turner National Medical Research Center for Children’s Orthopedics and Trauma Surgery, Pushkin, 196603 Saint Petersburg, Russia; anton-shab@yandex.ru; 3 Institute of Chemistry, Saint Petersburg State University, 198504 Saint Petersburg, Russia; 4 Institute of Silicate Chemistry, Russian Academy of Sciences, 199034 Saint Petersburg, Russia; dprom@mail.ru; 5 Institute of Theoretical and Experimental Biophysics, 142290 Pushchino, Russia; antonpopovleonid@gmail.com (A.L.P.); ao_ermakovy@rambler.ru (A.M.E.); 6 Kurnakov Institute of General and Inorganic Chemistry, Russian Academy of Sciences, 119991 Moscow, Russia; a.baranchikov@yandex.ru (A.E.B.); van@igic.ras.ru (V.K.I.); 7 Research Institute for Physical and Chemical Problems, Belarusian State University, 220030 Minsk, Belarus; sergeysolomevich@gmail.com (S.O.S.); bychkovsky@tut.by (P.M.B.)

**Keywords:** nanocomposites, bacterial cellulose, ceria nanoparticles, thermal properties, swelling, mechanical behavior, biomedical applications, stem cells proliferation, gene expression

## Abstract

A technique for the fabrication of bacterial cellulose-based films with CeO_2_ nanofiller has been developed. The structural and morphological characteristics of the materials have been studied, their thermal and mechanical properties in dry and swollen states having been determined. The preparation methodology makes it possible to obtain composites with a uniform distribution of nanoparticles. The catalytic effect of ceria, regarding the thermal oxidative destruction of cellulose, has been confirmed by TGA and DTA methods. An increase in CeO_2_ content led to an increase in the elastic modulus (a 1.27-fold increase caused by the introduction of 5 wt.% of the nanofiller into the polymer) and strength of the films. This effect is explained by the formation of additional links between polymer macro-chains via the nanoparticles’ surface. The materials fabricated were characterized by a limited ability to swell in water. Swelling caused a 20- to 30-fold reduction in the stiffness of the material, the mechanical properties of the films in a swollen state remaining germane to their practical use. The application of the composite films in cell engineering as substrates for the stem cells’ proliferation has been studied. The increase in CeO_2_ content in the films enhanced the proliferative activity of embryonic mouse stem cells. The cells cultured on the scaffold containing 5 wt.% of ceria demonstrated increased cell survival and migration activity. An analysis of gene expression confirmed improved cultivation conditions on CeO_2_-containing scaffolds.

## 1. Introduction

In the 21st century, there has been growing attention among researchers and chemical engineers in the field of novel polymer materials in so-called natural or “green” polymers, including cellulose and its derivatives [1]. Of particular interest are materials based on bacterial cellulose (BC) [2], which is a product of the vital activity of *Acetobacteraceae bacteria* (Gram-negative anaerobic bacteria) in carbon-containing nutrient media. Typically, the media are aqueous solutions of carbohydrates [2], mainly glucose, less commonly saccharose, fructose, maltose, xylose, and polyatomic alcohols, etc.

BC is a hydrophilic material with a high degree of structural ordering of macromolecules (the degree of crystallinity is as high as 90–95%). It possesses a complex hierarchical supramolecular structure which consists of ribbon-like fibrils up to 100 nm wide. The fibrils are formed of small nanofibrillar elements with a characteristic size of 7–10 nm assembled in bundles and tows [1,2].

BC does not contain any contaminants such as lignin, pectins, or hemicellulose, which are present in plant analogues of BC even after purification [2]. BC is characterized by high mechanical strength and stiffness, and the capability of retaining large amounts of water when swelling (up to 98–99% of the total mass of the material, depending on preparation technique). Moreover, it is biodegradable and has excellent biocompatibility, including with human tissues. BC has the merit of being a non-cytotoxic, non-genotoxic material, causing no allergic reactions from a human organism. For these reasons BC seems to be an extremely promising material for biomedical applications [3,4,5,6].

The great scope for the development and further modification of “green” materials in order to improve their characteristics is associated with the preparation of composites based on BC with either inorganic or organic components [7,8,9,10], particularly with nanoparticles. Such an approach is widely used in the fabrication of novel polymer-based materials. Oxide nanoparticles are among the most commonly employed active nanofillers used for the enhancement of polymer properties. Nanoparticles have been proven to substantially alter various properties of materials, such as their mechanical, thermal, electrical, and transport properties [11,12,13,14]. Therefore, metal oxide nanoparticles are likely to have a profound effect on a number of cellulose functional properties.

Cerium oxide (ceria) nanoparticles are promising for use as an active nanofiller in polymer composites [15]. They have been shown to solve the following problems in polymer science: the screening of electromagnetic irradiation, fabrication of catalysts, modification of the acoustic properties of a polymer matrix, etc. [16,17,18,19,20,21]. Last but not least, the possibility of using nanosized CeO_2_ in biomedical nanocomposite materials seems quite promising, taking into account a great deal of information on the salient biological activity of ceria. The latter has been reported as an antibacterial [22], antioxidant [23,24], and antitumour [25,26] agent. Ceria has been demonstrated to prevent sepsis [27]. The positive effect of nanosized cerium dioxide on the intensity of proliferation of stem cells on CeO_2_-containing scaffolds has been observed in a number of works [28,29]. The most recent advances in properties and applications of nanoceria are summarized elsewhere [30]. Thus, ceria-containing polymer nanocomposites are anticipated to combine the advantageous properties exhibited by both the matrix and the filler and to reduce their intrinsic drawbacks.

This paper reports on what was intended to be a thorough investigation of the effect of ceria as an active nanofiller on the characteristics of BC-based nanocomposite materials. Their biological activity has been tested, with a focus on their use in bioengineering, specifically as scaffolds for stem cell proliferation. It has already been shown that bare BC can be used as a substrate facilitating tissue regeneration and possessing cell affinity [31,32,33]. Therefore, it is reasonable to assess the effect of CeO_2_ nanoparticles on cell proliferation on BC-based scaffolds.

## 2. Materials and Methods

### 2.1. Reagents

Glucose (≥99.5%, #G7021), ethanol (96.0–97.2%, #24105), citric acid (≥99.5%, Sigma-Aldrich, #251275), and cerium (III) nitrate (99%, #238538) were purchased from Sigma Aldrich. Chemically pure yeast extract was provided by “best Group” (Moscow, Russia). Isopropanol (≥99.8%, puriss p.a.) was purchased from Khimmed (Moscow, Russia).

### 2.2. Bacterial Cellulose

BC was produced by *Kommagateibacter xylinus* (acetic acid bacteria, a VKM-880 strain) in aqueous solutions containing 2 wt.% of glucose, 0.3 wt.% of yeast extract, and 2 wt.% of ethanol at 30 °C for 14–21 days [34]. The as-prepared BC samples were gel-like pellicles up to 25 mm thick. The pellicles were partially dehydrated using a hand press to fabricate BC films, the BC concentration in the films being about 14 wt.%.

### 2.3. CeO_2_ Nanoparticles

The cerium dioxide nanoparticles, with sizes ~3.5–5 nm, used in the experiments were synthesized using a modified protocol, reported elsewhere [35]. Briefly, to a water-isopropanol (1:19 vol.) 0.1 M solution of cerium(III) nitrate, a water-isopropanol (1:5 vol.) solution of citric acid (cerium to citrate ratio 1:1) was added to form a white sediment. The resulting powder was thoroughly washed, dried, and then dispersed in distilled water. Concentrated ammonia solution was added to a suspension and the mixture was refluxed in air at pH 9–10 for 12 h. The resulting ceria sol was boiled for 1 h to remove any excess of ammonia, to pH 8.

### 2.4. Thin Film Fabrication

Pressed BC was fragmented in a high-speed blender (15,000 rpm, 15 min) in an aqueous medium (300 mL of water per 1 g of dry BC). The suspension prepared was cast onto a glass support and dried at 160 °C to a constant weight. In order to prepare the nanocomposites, certain amounts of ceria aqueous dispersions (with CeO_2_ concentration of 1.5%) were poured into the BC aqueous suspension. The films were fabricated using the aforementioned technique. All the films prepared were dense, homogeneous and ~40 μm thick. The nominal concentrations of CeO_2_ nanoparticles in the samples were 0 wt.% (bare BC film), 1 wt.%, 3 wt.%, and 5 wt.%.

### 2.5. Film Characterisation Techniques

#### XRD Analysis

The X-ray diffraction (XRD) analysis of the films was performed on a DRON-3M X-ray diffractometer (CuKα-irradiation). The registration of X-ray beam diffraction was carried out according to the Bragg–Brentano scheme [36].

### 2.6. Scanning Electron Microscopy

The surface of the films was studied using scanning electron microscopy (SEM). SEM images were obtained with a scanning electron microscope SUPRA-55VP (Carl Zeiss, Oberkochen, Germany), using a secondary electron detector, as well as a detector of back-scattered electrons. The samples were fixed with special glue on the microscope holders and sputtered by thin layer of platinum. Element maps were collected with an EDX-max 80 mm^2^ detector (Oxford Instruments, Oxford, UK).

### 2.7. Thermal Analysis

Thermogravimetric (TGA) and differential thermal (DTA) analyses were performed to determine the concentration of remaining water in the films and the exact contents of ceria in the nanocomposite materials. The effect of CeO_2_ on the kinetics of thermal decomposition of the samples was assessed. A DTG-60 thermal analyser (Shimadzu, Kyoto, Japan) was used, the samples (~5 mg) being heated in air up to 600 °C at a rate of 5 °C/min.

### 2.8. Swelling Properties

To determine equilibrium water absorption, the samples, having first been dried at 150 °C, were weighed and then immersed in the distilled water. The swollen samples were periodically removed from the water, wiped with a tissue, and weighed. The experiment continued until a constant weight had been attained.

Water absorption *W_m_* was calculated using the following equation:

$$W_{m}=\frac{m_{s}-m_{d}}{m_{d}}\;\times \;100,$$

where *m_s_* and *m_d_* are the weights of the swollen and dry samples, respectively.

### 2.9. Mechanical Properties

An AG-100kNX Plus setup (Shimadzu, Kyoto, Japan) operating in a uniaxial extension mode was used to study the mechanical characteristics of the films. Strip-like samples (2 × 30 mm^2^) were stretched at room temperature at a rate of 10 mm/min, according to ASTM D638 requirements. The Young’s modulus *E*, the break stress *σ_b_*, and the ultimate deformation *ε_b_* were determined.

### 2.10. Cell Proliferation Control Techniques

#### Cell Culture

The experiments were conducted using mouse mesenchymal stem cells (MSC) at the 3rd passage, harvested from the embryonic skeleton of 13 day-old embryos of B10GFP/Balb/c hybrid mice carrying a green fluorescent protein (GFP). The cells were received from the vivarium of ICB RAS (Pushchino, Moscow Region, Russia). The study was conducted according to the guidelines of EU directive 2010/63 of European Parliament and council of Europe Union 22 September 2010 for the protection of animals used for scientific purposes, and approved by the Institutional Ethics and biosafety committee of Institute of Theoretical and Experimental Biophysics Russian Academy of Sciences. The animals were fed twice a day. Animal maintenance was in accordance with the rules of Good Laboratory Practice (GLP) and the Order of the Ministry of Health of the Russian Federation No. 199n “Rules of Good Laboratory Practice”.

Using this type of cell was regarded as being expedient due to the need to avoid applying fluorescent dyes in order to prevent the coloration of the samples under study. Mice were euthanized by cervical dislocation on the 13th day of gestation. Their uteri with embryos were isolated. After tissue dissociation in 0.25% trypsin-0.02% EDTA solution (NPO “PanEco”, Moscow, Russia) for 30 min at 37 °C, the cells were collected by centrifugation at 1500 rpm for 2 min. Then, the cells were re-suspended to obtain a single-cell state in a DMEM/F12 medium (NPO “PanEco”, Russia) supplemented with 10% of fetal bovine serum (FBS) (in 1:1 ratio). The resulting suspensions were transferred into vials and cultivated in a 5% CO2 atmosphere at 37 °C in DMEM containing 10% FBS (HyClone, Logan, MI, USA) and 100 units/mL of penicillin/streptomycin, with the addition of 2 mM L-glutamine. When a subconfluent state was reached, the cells were treated with 0.25% trypsin-EDTA solution and seeded into vials in a 1:3 ratio. The cultivation was carried out in a DMEM/F12 medium containing 10% FBS (HyClone, Logan, MI, USA) and 100 units/mL of penicillin/streptomycin, with the addition of 2 mM L-glutamine. Cultures of 2–5 passages were used in the research. The analysis of the viability of cell cultures was carried out using the resazurin assay. After 24–96 h of cultivation, the cell culture medium was replaced with one containing 0.02% solution resazurin and the MSC were further cultured.

### 2.11. Fluorescent Microscopy

The films were cut into 0.8 × 0.8 cm^2^ fragments and placed in the wells of a 24-well plate, under sterile conditions. One sample was assigned to each test point. A sterile cover glass was used as a reference sample. Cells were seeded on the substrates’ surface. The initial cell seeding density was 30 × 10^4^ cells/cm^2^. The morphological assessment of the cells cultivated on the surface of the test materials was implemented on the 1st, 2nd, 3rd, and 4th day of cultivation in fluorescent light, using an inverted microscope Zeiss Axiovert 200 (Carl Zeiss, Oberkochen, Germany).

### 2.12. Scanning Electron Microscopy

The cells were plated on the substrates and cultivated for 24 to 96 h. The samples were then washed twice for 30 min in phosphate-buffered saline (PBS) at pH 7.4 and dehydrated by being passed through alcohol solution of increasing concentration: 30%, 50%, 70%, and 80% for 30 min, and 90% and 100% for 30 min (twice). At the final stage, the samples were placed in a hexamethyldisilazane solution for 24 h. In order to avoid charge accumulation during scanning, the samples were coated with a 2 nm gold layer by ion sputtering in an argon atmosphere (0.1 mm Hg). A SUPRA-55VP (Carl Zeiss, Oberkochen, Germany) microscope was used in the experiments.

### 2.13. Reverse Transcription Polymerase Chain Reaction (PCR-RT)

Reverse transcription was performed with a Sileks kit (Sileks, Moscow, Russia), using an oligo(dT) primer, according to the manufacturer’s protocol. The cDNAs produced served as a real-time PCR matrix. For PCR reaction, a mixture was used with SYBR Green dye (Syntol, Moscow, Russia). To analyze the expression activity of 93 key genes under cell cultivation on the CeO_2_-containing scaffolds, a CFX-96 amplifier ((BioRad, Irvin, CA, USA) or an ABI 7500 Fast Real-Time PCR System (Applied Biosystems, Waltham, MA, USA) was used. The expression of 96 genes responsible for key cell processes (Supplementary Table S1) was thus determined. The genes that were analyzed were selected from the database https://dataanalysis2.qiagen.com/pcr (accessed on 16 June 2021) for PCR profiling of different biological processes. The level of gene transcription was normalized according to the levels of expression of housekeeping genes *β-actin*, *rplp0* (ribosomal protein, large, P0), and *gapdh* (glyceraldehyde-3-phosphate dehydrogenase). Gene-specific primers were selected using the Primer Express program (Applied Biosystems, Waltham, MA, USA). Each measurement was made twice (internal repetition) and averaged for two independent samples. A sample without a reverse transcription stage was used as the control. The expression data obtained were analyzed using online services https://dataanalysis2.qiagen.com/pcr, mayday-2.14 software (Center for Bioinformatics, Tübingen, Germany) and Genesis software.

## 3. Results

### 3.1. SEM

**Figure 1:**
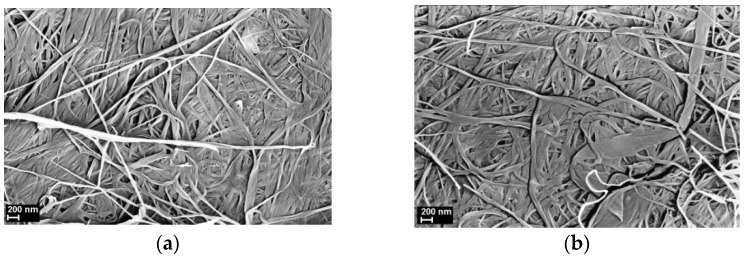

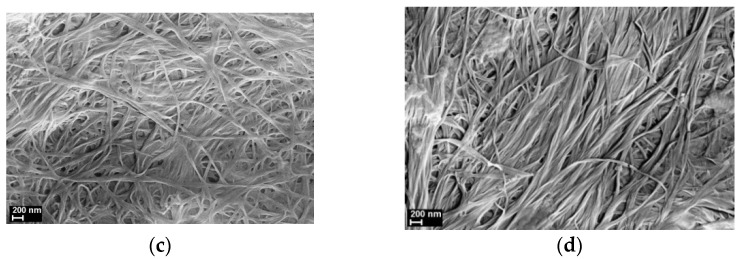
SEM images of the samples studied: (**a**) bare BC; (**b**–**d**) BC-ceria nanocomposite films containing 1 wt.%, 3 wt.%, and 5 wt.% CeO_2_, respectively.

SEM images of the films fabricated (Figure 1) show clearly the structural elements of the films, namely tightly-packed fibrils. Comparing the morphologies of the composite and pristine BC films, there is no noticeable effect of CeO_2_ nanoparticles on the packing type, except that the BC fibrils in the composite films seem to be aggregated more closely. No evident aggregates of CeO_2_ nanoparticles are seen on the composite films’ surfaces.

### 3.2. XRD Analysis

**Figure 2:**
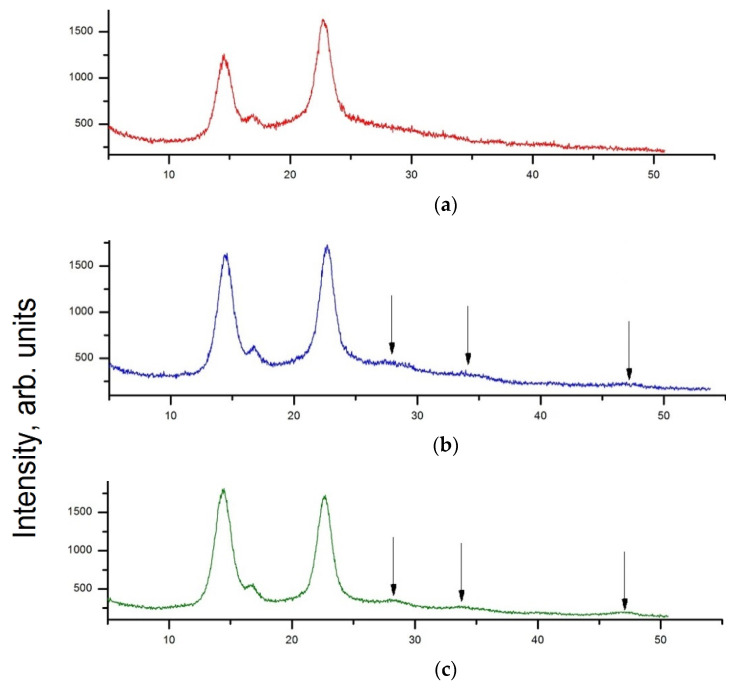

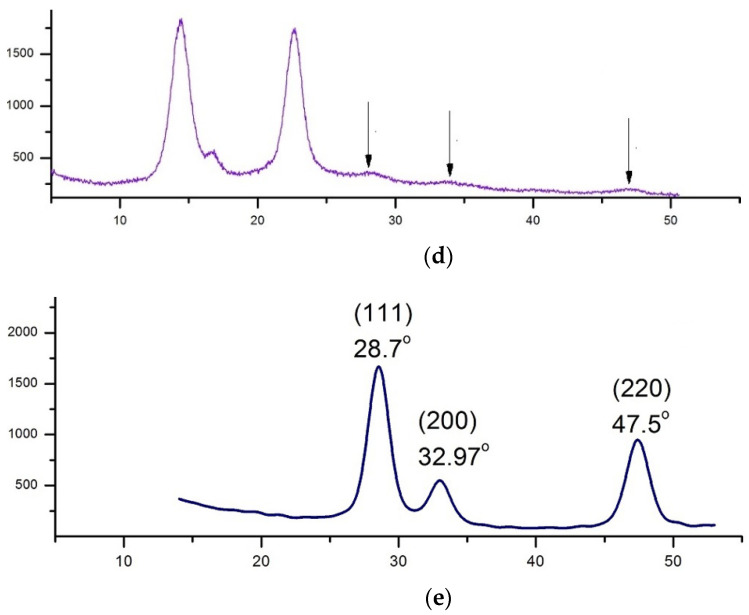
XRD patterns of the films: (**a**) bare BC; (**b**–**d**) BC-ceria nanocomposites with 1 wt.%, 3 wt.%, and 5 wt.%, respectively, (**e**) X-ray diffraction pattern of ceria nanoparticles. Arrows in XRD patterns of the composite samples indicate CeO_2_ reflections.

Obviously, the SEM technique does not enable individual filler nanoparticles to be distinguished. Their presence in the films is only evident from the comparative analysis of the XRD patterns of the films and pristine CeO_2_ powder (Figure 2). Well-resolved reflections at 2θ near 14.4°, 16.7°, and 22.4° are revealed in all XRD patterns of BC-based films. According to the data of X-ray scattering, the structure of these specimens is attributed to highly crystalline cellulose I, most probably Iα allomorph [37].

The XRD pattern for CeO_2_ powder shows sharp reflections at 2θ = 28.7, 32.97, and 47.5 deg. attributed to (111), (200), and (220) planes, respectively, corresponding to the cubic fluorite crystal structure (ICDD PDF card #34-394, data from NIST (National Institute of Standards and Technology, Gaithersburg, Maryland, Boulder, CO, USA)). The full width at half-maximum (FWHM) of the (111) peak was used in the calculation of the apparent transverse D111 sizes of CeO_2_ crystals, using Scherrer’s equation [38]. The D_111_ size was found to be ~5.4 nm. The same reflections, along with those of the BC, can be seen in the patterns of the nanocomposite films (Figure 2b–d). Their intensities increased linearly with the concentration of ceria nanoparticles in the BC matrix.

### 3.3. EDX Analysis

**Figure 3:**
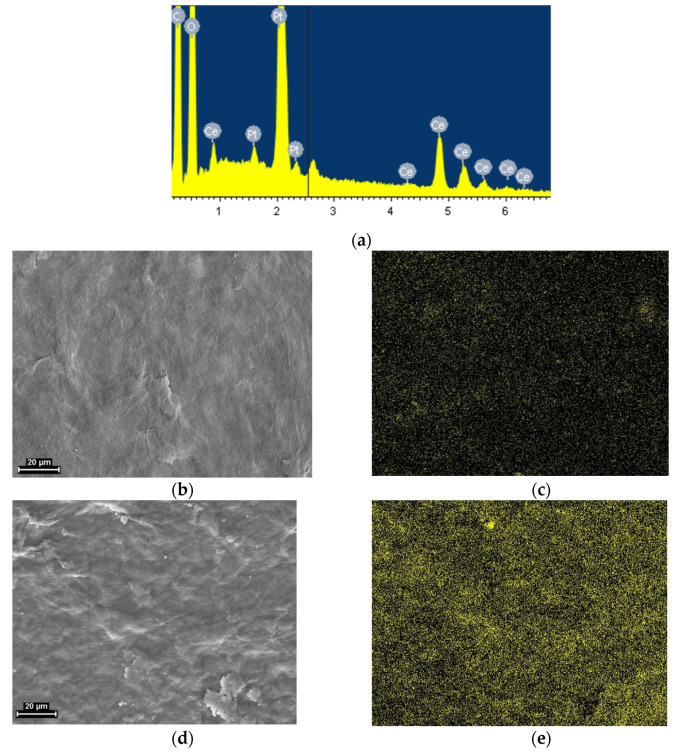
(**a**) An example of the EDX spectrum of the composite BC-CeO_2_ sample; SEM images (left side) and EDX maps of cerium distribution (right side) on the surface of the BC-ceria nanocomposite samples with: (**b**,**c**) 1 wt.% CeO_2_; (**d**,**e**) 5 wt.% CeO_2_.

Figure 3 shows the results of energy dispersive X-ray analysis (EDX). This method enables the distribution of elements on the surface of a sample to be mapped. The results confirm the presence and increasing concentration of cerium (the areas appearing as speckled yellow in Figure 3c,e) in the samples, images of the latter being shown in Figure 3b,d. The information on the total percentage of Ce can also be obtained by EDX analysis. It was derived that the weight amounts of Ce are the following: 0.81 wt.% for the composite film containing 1% CeO_2_, and 4.02 wt.% for the 5% CeO_2_ film.

### 3.4. Thermal Analysis

**Figure 4:**
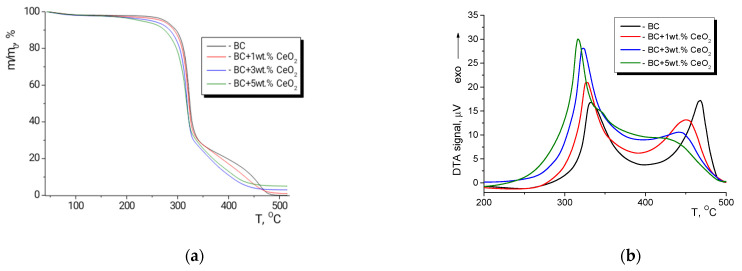
(**a**) TGA and (**b**) DTA curves of bare BC film and nanocomposite films containing different amounts of ceria.

TGA was used to precisely determine the nanofiller concentration in the nanocomposite films prepared (Figure 4). The shape of the BC weight loss curve in air (Figure 4a) is attributable to the complete decomposition of the polymer upon heating to 500 °C, accompanied by the removal of the gaseous products. A different outcome is observed from the TGA of the composite films (Figure 4a), viz., after total volatilization of the organic part of the material, stable inorganic residues were obtained, whose concentrations in the films (1.0 wt.%, 3.05 wt.%, and 4.90 wt.%) corresponded to those used in the fabrication of the composite samples.

The TGA curves also enable an evaluation of the weight of water remaining in the as-prepared films. The curves show an initial weight loss step below 120 °C, followed by a plateau at higher temperatures, where the weight did not change until the start of thermal decomposition. The weight loss in all of the samples in this low-temperature region was ~2.0–2.5%. The TGA curves corroborate the literature data [18,39] on the catalytic activity of ceria in decomposition reactions of a number of organic compounds. Comparing the TGA results for the pristine BC film and the composites with various CeO_2_ contents, it is noticeable that intense decomposition processes shifted to lower temperatures as ceria concentration increased. The deterioration in the thermal stability of the samples was accompanied by a decrease in the indices τ_5_ and τ_10_ (the temperature values at which a polymer or a composite loses 5% and 10% of its initial weight, respectively, due to thermal destruction processes) (Table 1).

A detrimental effect of CeO_2_ on the thermal stability of BC is evident from the DTA curves of the nanocomposite films (Figure 4b). Indeed, an increase in ceria concentration from 0 to 5 wt.% led to a gradual shift of exothermic peaks toward the low-temperature region, the peaks being ascribed to the heat effect of thermal destruction. In accordance with previously reported data [40,41], the thermo-oxidative destruction of BC and BC-based nanocomposites occurred in two steps (two peaks on the DTA curves corresponding to these steps are in the 300–330 °C and 420–480 °C temperature regions). The increase in ceria content is seen to result in both a shift of the exothermic peaks to lower temperatures and a redistribution of the peak heights: a low-temperature peak became higher, whilst a high-temperature peak tended to become lower and smoother. In accordance with the well-known scheme of cellulose destruction—the Broido–Shafizadeh model [42]—the main reaction taking place in the low-temperature region (up to 350 °C) is the thermally stimulated decomposition of cellulose macrochains, resulting in the formation of monomeric and oligomeric fragments (the so-called “sirup” fraction). At the next (high-temperature) stage, these fragments decompose further, producing a variety of gaseous products. Based on the results obtained in the experiments on which this paper reports, it can be inferred that CeO_2_ causes deep depolymerization of BC, and full decomposition of the oligomers into monomers during the first stage of the destruction, thus facilitating the second stage.

It is worth mentioning that all of the aforementioned processes start at temperatures higher than 180–200 °C, and this should be taken into account when thermally sterilizing nanocomposite BC-ceria materials for biomedical applications.

### 3.5. Mechanical Properties

The successful practical use of the composite materials devised requires certain mechanical characteristics of the films they form. The mechanical properties of the BC-ceria composite materials with different CeO_2_ content are summed up in Table 2.

The data given in Table 2 reveal both the high stiffness (Young’s moduli of all the materials are higher than 5 GPa) and good strength characteristics (mechanical strength reaches 100–110 MPa) of the films. The incorporation of ceria in the BC matrix is seen to lead to a gradual (as CeO_2_ concentration grows) increase in the elastic modulus (5 wt.% of CeO_2_ augment the modulus by 27%). Such a pronounced rise in stiffness cannot be attributed to the reinforcing action of the nanofiller on BC matrix, as CeO_2_ nanoparticles, which have quasi-spherical shapes (aspect ratio is ~1) and are taken in rather small amounts, do not provide a reinforcing effect. A sensible explanation is that additional links between the BC macrochains form via the active surface of the nanoparticles when the nanocomposite is fabricated. It should also be pointed out that ceria does not cause a substantial decrease in the ultimate deformation of the films. This confirms the good compatibility between both components of the nanocomposites.

### 3.6. Ability to Swell in Water

The discussion above concerns the mechanical properties of dry films (according to TGA, water content is ~2.0–2.5%). However, the capability of a material to absorb water is of crucial importance in a number of practical applications, including biomedicine. Therefore, the current study considered the impact of ceria upon this property of the BC films.

**Figure 5:**
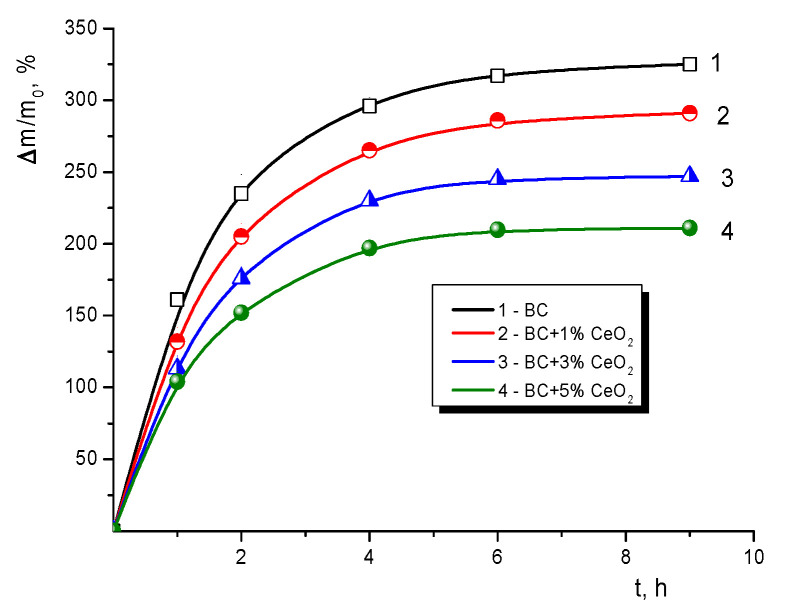
Swelling kinetics of bare BC and BC-CeO_2_ nanocomposite films.

It is evident from the swelling curves (Figure 5) that the films demonstrated a limited ability to swell in aqueous media, viz. the ultimate amount of water in the matrix BC in a swollen state was only 3.25 times higher than the weight of the dry polymer. Apparently, such a limited ability to swell is determined by the characteristics of the intermolecular bonds formed during fabrication of the films.

A fairly interesting phenomenon observed in the experiments was the fall in the ultimate amount of water in a swollen material as CeO_2_ content increased (Figure 5). This observation corroborates the hypothesis about additional intermolecular interactions, the CeO_2_ nanoparticles acting as links. A similar reduction in the degree of swelling in water was observed in nanocomposite films consisting of a mixture of chitosan with cellulose acetate filled with CeO_2_ nanoparticles [43].

An assessment of the mechanical properties of the swollen films is important in the context of the practical use of the materials. A technique was developed for performing mechanical tests on the swollen samples. Their mechanical characteristics are shown in Table 2. The results show explicitly that, even in an extremely swollen state, the films possessed a certain mechanical rigidity. The elastic moduli of the samples fell to 0.03–0.04 of that of a dry film but were of the same order of magnitude as the modulus of the LDPE film (170–300 MPa). It should be pointed out that a less pronounced decrease in the elastic modulus caused by swelling was registered in the nanocomposite films in comparison with the pristine BC sample. Such a trend was observed as ceria concentration in the material was augmented: the ratio of the elastic modulus of a film in a fully swollen state, to that of a dry film increased from 0.032 to 0.044 when the CeO_2_ content changed from 0 to 5 wt.% (Table 2). This clearly reflects a gradual decrease in the degree of swelling of the films with a rise in the concentration of the nanoparticles in the BC matrix. Despite a fairly significant drop in stiffness, the swollen films were characterized by a mechanical strength of 4–6 MPa, the strength being high enough for the material to be used in some biomedical applications.

### 3.7. MSC Proliferation

Among the possible applications of BC-ceria nanocomposite materials, their use in bioengineering as scaffolds for the proliferation of stem cells seems to be very promising. One of the main, and most auspicious, prospects for steady progress in modern medicine is associated with the development, and improvement, of the technology for growing cells of this kind. BC is known to be an interesting and promising material in the manufacture of such substrates [31,32,33,44]. This is due to the peculiarities of the structure of the material, its excellent biocompatibility combined with its high bioresorption ability, its non-cytotoxicity, and the possibility of its preparation in an extra-pure state. There were good reasons to combine such merits of BC matrices with those inherent in ceria in biological systems.

**Figure 6:**
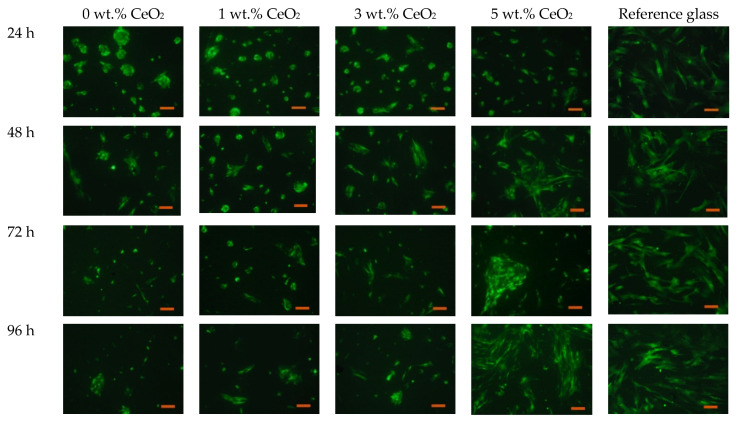
Fluorescence microscopy images of mouse MSC with GFP gene on the surface of the substrates.

Experiments were carried out on MSC proliferation on substrates made of bare BC and nanocomposites containing up to 5 wt.% of CeO_2_. Figure 6 shows the data on mouse MSC proliferation activity on the surface of bacterial cellulose, the latter containing various concentrations of CeO_2_ nanoparticles. The results were recorded on the 1st, 2nd, 3rd, and 4th day of cultivation under fluorescent light.

The CeO_2_-containing scaffolds demonstrated good adhesion with the mouse MSC. It is well known that the adhesion of cells to the scaffold surface is strongly affected by the micro-relief and texture of the latter [45]. The addition of CeO_2_ nanoparticles to the BC matrix is supposed to create a surface structure that is most suitable for the formation of focal contacts of the cell filopodia, followed by the protrusion of the cell front. A certain electric charge on the scaffold surface also ensures optimal conditions for proliferation [43]. In the case of BC-CeO_2_ nanocomposite materials, the electric charge might be provided by the nanoparticles [46].

The adhesive characteristics of the composite scaffolds differ considerably, depending on ceria content. The minimum concentration of the nanoparticles (1 wt.%) provided adhesion for a substantial part of the cell culture; however, after 24 h, morphological features of the cells differed from those on the reference substrate. In particular, the cells did not exhibit the characteristic protrusions and formed multicellular aggregates, whose size decreased with an increase in ceria concentration in the scaffold. This might be explained by weak surface micro-relief, as well as by insufficient adhesion in initial sites for the formation of a fully-fledged extracellular matrix. Further cultivation on the scaffolds demonstrated that the cells spread effectively on the substrate even at small CeO_2_ concentrations. This process, however, took longer than that on the scaffolds with 3 and 5 wt.% of ceria.

It should be pointed out that the scaffolds with high concentrations of CeO_2_ nanoparticles (3 and 5 wt.%) exhibited notably higher cell viability after 96 h of cultivation than the scaffolds with 0 and 1 wt.% of the nanoparticles. The quantity of cells on these scaffolds was also substantially higher than that of the reference sample (cover glass). Thus, it can be inferred that ceria nanoparticles embedded in a scaffold ensure an increase in the rate of mouse MSC proliferation. This confirms the stimulating action of the hybrid scaffolds in comparison with those with no ceria. No difference was observed between the substrates with high CeO_2_ concentrations and the reference sample, at the early stages of cultivation (12 and 24 h), but longer cultivation periods demonstrated the well-defined stimulating effect of the former.

**Figure 7:**
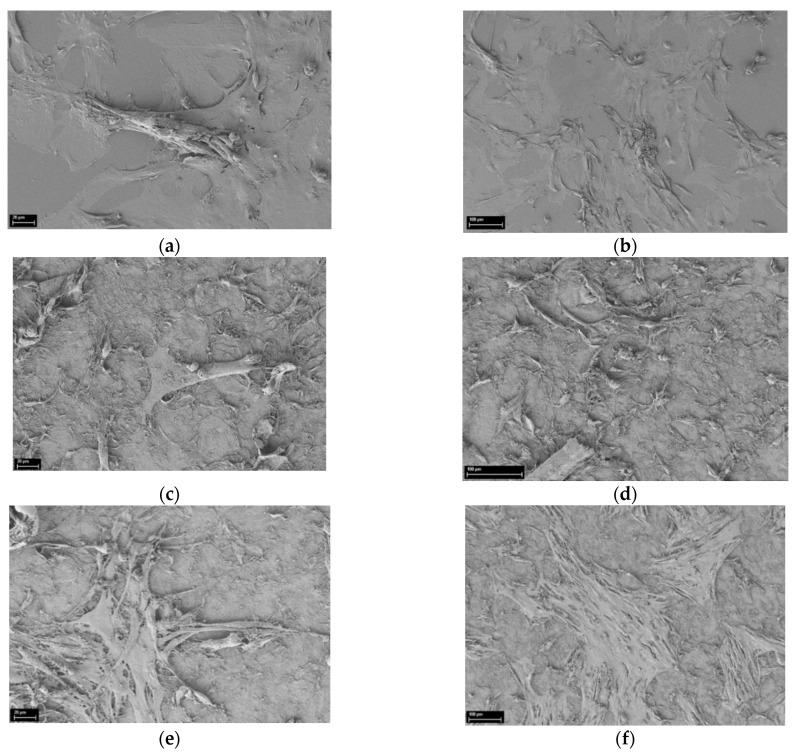
SEM images of the scaffolds after 96 h of mouse mesenchymal stem cells cultivation. (**a**,**b**) The reference sample (cover glass); (**c**,**d**) bare BC film; and (**e**,**f**) BC with 5 wt.% of CeO_2_ nanoparticles.

SEM images of the mouse MSC on the surface of BC film, with maximal content of CeO_2_ nanoparticles on the 4th day of cultivation, are shown in Figure 7, and can be compared to those cultivated on bare BC and glass substrate.

Analysis of the SEM images reveals the developed surface microstructure of the scaffolds. Long-term cultivation (96 h) resulted in migration and proliferation activity of the mouse MSC on the hybrid CeO_2_-containing substrates. This is evident from the formation of more focal contacts, as well as from an increase in the protrusion area. The cell monolayer formed on the BC substrates with high CeO_2_ concentrations (3–5 wt.%) had morphological features typical of actively dividing cells, these features being less pronounced for scaffolds containing 0 and 1 wt.% of ceria.

**Figure 8:**
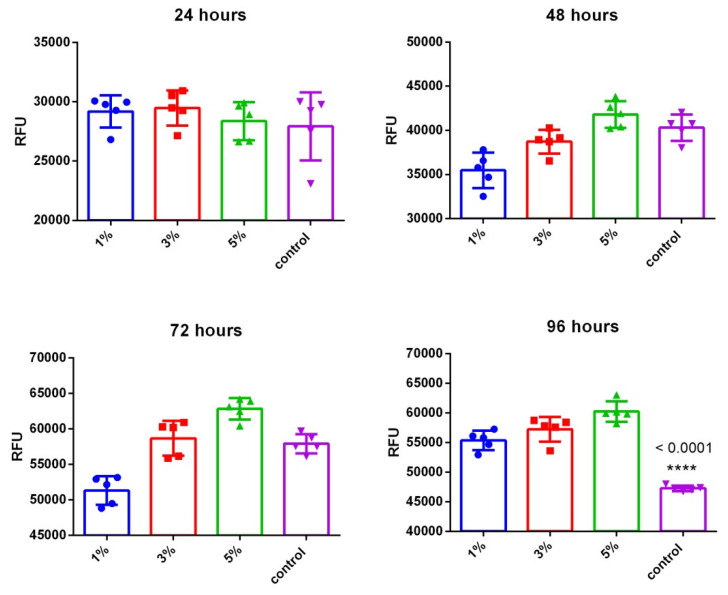
Viability of mouse MSC culture after cultivation on CeO_2_-containing BC scaffolds for 96 h. Data are presented as the mean value ± SD. Cover glass was used as a control. **** *p* < 0.0001 via control.

Quantitative analysis of the number of cells adhered to CeO_2_-containing scaffolds showed that all the scaffolds provided effective cell adhesion. The analysis of proliferative activity was carried out during four days of cultivation by resazurin test (Figure 8). After 48 h of cultivation, the CeO_2_-containing scaffolds provided a significant increase in the number of cells, which was confirmed by increased values of resazurin fluorescence. The results obtained show a dose-dependent effect of ceria-containing substrates on cell culture proliferation.

**Figure 9:**
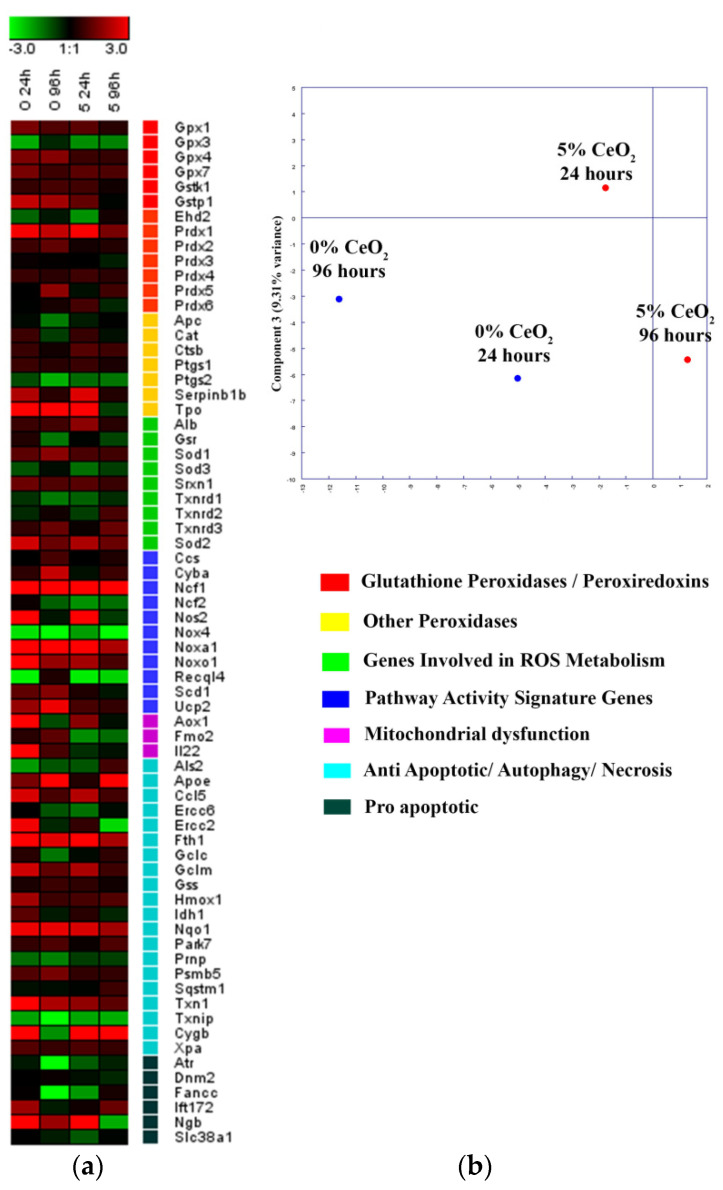
Heat map of gene expression in mouse MSC, cultured on 0% and 5% CeO_2_-containing scaffolds, after 24 and 96 h of cultivation. (**a**) The intensity scale of the standardized expression values ranges from −3 (green: low expression) to +3 (red: high expression, with 1:1 intensity value (black) representing the control (nontreated). Principal component analysis (PCA) of qRT-PCR data for different concentrations of CeO_2_ scaffold (**b**). Cluster groups of genes and their functionality.

The molecular mechanisms of stimulation of MSCs proliferation in the presence of cerium dioxide nanoparticles are associated with the regulation of MSCs redox status in particular; nanoceria reduces the level of intracellular reactive oxygen species (ROS), and a high ROS concentration inhibits the rate of proliferation. Most likely, biological activity of nanoceria with regard to MSCs is connected to mixed cerium valence state in cerium dioxide nanoparticles [29]. For example, nanoceria has been shown to possess anti-inflammatory properties [47]; the treatment with nanoceria reduced both the level of IL-6 and inflammatory markers in the blood of mice in both in vitro and in vivo experiments [27]. Enhanced bone regeneration due to the activation the ERK signaling pathway by nanoceria has been also reported [28]. In our manuscript, using the PCR-RT, we confirm the contribution of cerium oxide to the acceleration of the MSCs proliferation through the regulation of genes associated with antioxidant enzymes (Figure 9a).

The polymerase chain reaction method (PCR-RT) was used to study the expression of gene clusters (panel of 93 genes) encoding antioxidant enzymes (the family of glutathione peroxidases and peroxiredoxins) and genes involved in the regulation of ROS methabolism (SOD-1, SOD-2, catalase, and thioredoxins, etc.). We also analyzed the genes responsible for mitochondrial dysfunction, as this is the main cellular organoid involved in ROS formation. These genes are responsible for the activation of a number of cascade pathways, including apoptosis, necrosis, antioxidant system, differentiation, proliferative and migration activities, the repair system, and pro-inflammatory response. PCR-RT was used to study the cells cultured on substrates containing 0 and 5% CeO_2_ (Figure 9). After 24 h of cultivation, activation was observed for peroxiredoxin groups, and genes PRDX1, NCF1, Nos2, Tro, Noxo1, Noxa1, AOX1, FTH1, Ngb, and Nqo1. After 96 h of cultivation, NCF1, Tro, Noxa1, FTH1, and Nqo1 also retained a high level of expression. According to PCA analysis (Figure 9b), ceria-containing substrates demonstrated a significant difference in gene expression pattern in comparison with ceria-free substrates. The CeO_2_-containing scaffolds significantly increased the integral index of the upregulation in the expression pattern of the selected genes, which indicates a positive effect of ceria-modified scaffolds for the metabolic microenvironment and MSCs redox status.

To summarize, it can be inferred that MSC proliferation proceeds more effectively on the scaffold with the highest CeO_2_ concentration used in the study, i.e., 5 wt.%. Such a scaffold is characterized by a water absorption value that is high enough to ensure nutrient exchange capacity, and that is sufficient to enable intense proliferation. It may be assumed that a further increase in ceria content would facilitate a stimulating activity of the scaffold, its high biodegradability, and the maintenance of cytoneutrality.

## 4. Conclusions

A method of fabrication of BC-CeO_2_ composite films has been proposed and implemented to prepare biologically active materials capable of enduring mechanical stress. A combination of XRD and SEM analyses corroborated the uniform distribution of the nanoparticles in the BC matrix. Simultaneous TGA and DTA revealed the pronounced catalytic activity of ceria regarding the thermo-oxidative destruction processes in BC-based nanocomposites.

The materials obtained exhibited a limited ability to swell in an aqueous medium, the addition of ceria to the BC matrix causing a decrease in the equilibrium concentration of water in the swollen films. The water content changed from 325 to 210% of a dry sample’s mass with a change from the bare BC to the film with 5 wt.% of ceria.

CeO_2_ nanoparticles imparted BC films with enhanced stiffness, the maximum value of the latter being observed in the nanocomposite with 5 wt.% of the filler (27% higher than that of the unfilled BC film). Such an increase in stiffness of the material, along with a decrease in its ability to swell, points to the additional intermolecular interactions via the active surfaces of CeO_2_ nanoparticles. The swollen films have a stiffness that is equal to 0.03–0.04 of that of the dry films, the elastic moduli and strength values being 170–300 MPa and 4–6 MPa, respectively. Such characteristics are similar to those of a number of human tissues, such as muscles, cartilages, and ligaments [48].

It was concluded, after examination of the nanocomposite materials regarding their use as bioresorbable scaffolds for the mouse MSC proliferation, that ceria positively affected the cells’ attachment to a substrate, followed by their growth. The ceria-containing scaffolds possess a high degree of adhesion with regard to the stem cells via the formation of the focal contact of cell filopodia on their surface. The scaffold with the highest CeO_2_ concentration (5 wt.%) demonstrated the best cell viability after 96 h of cultivation. A cell monolayer formed on this substrate had morphological features typical of an actively dividing culture. The number of cells on the composite substrates was considerably higher than that on a reference sample (glass substrate). Thus, it can be inferred that the stem cells proliferate faster in the presence of CeO_2_ nanoparticles. This confirms the stimulating effect of the hybrid substrates compared to the bare BC. The nanocomposite sample with 5 wt.% of ceria was characterized by a water absorption value that is considered to be high enough to provide the good nutrient exchange capacity needed for intensive cell proliferation. BC-based composites containing 5% CeO_2_ provided upregulation of the main clusters of genes responsible for proliferation, migration, metabolism of reactive oxygen species, and downregulation of proapoptotic, proinflammatory and mitochondrial dysfunction genes.

## Figures and Tables

**Table 1:** Thermal stability indices of the BC and BC-ceria nanocomposite films.

Sample	τ_5_, °C	τ_10_, °C
BC	286	302
BC + 1 wt.%CeO_2_	283	298
BC + 3 wt.%CeO_2_	272	291
BC + 5 wt.% CeO_2_	261	281

**Table 2:** Mechanical characteristics of bare BC and BC-CeO_2_ composite films.

Composition	E, GPa	E_swollen_/E_dry_^1^	σ_b_, MPa	ε_b_, %
BC	5.5 ± 0.3		101 ± 5	3.6 ± 0.2
BC, swollen	0.17 ± 0.02	0.032	4.2 ± 0.4	2.9 ± 0.2
BC + 1 wt.% CeO_2_	5.8 ± 0.3		96 ± 3	2.7 ± 0.2
BC + 1 wt.% CeO_2_, swollen	0.20 ± 0.02	0.034	3.6 ± 0.3	2.6 ± 0.3
BC + 3 wt.% CeO_2_	6.3 ± 0.3		107 ± 3	3.3 ± 0.2
BC + 3 wt.% CeO_2_, swollen	0.24 ± 0.02	0.038	4.4 ± 0.4	2.2 ± 0.1
BC + 5 wt.% CeO_2_	6.9 ± 0.4		113 ± 5	3.3 ± 0.2
BC + 5 wt.% CeO_2_, swollen	0.30 ± 0.03	0.044	5.8 ± 0.3	2.8 ± 0.2

^1^ E_swollen_/E_dry_ is the ratio of the elastic modulus of a film with a given composition in a fully swollen state, to that of a dry film.

## Data Availability

Data sharing not applicable. No previously reported data were analyzed in this study. Data sharing is not applicable to this article.

## Supplementary Materials

The following are available online at https://www.mdpi.com/article/10.3390/polym13121999/s1, Table S1: Selected gene groups for PCR-RT analysis.
