# Supplementary material for: Bacterial Cellulose-Based Nanocomposites Containing Ceria and Their Use in the Process of Stem Cell Proliferation
Source: Polymers (Basel). 2021 Jun 18;13(12):1999. doi: 10.3390/polym13121999 (PMC8234971; doi:10.3390/polym13121999)
Supplement: Supplementary file 1 [file polymers-13-01999-s001.zip › polymers-1248690-supplementary.pdf]

Supplementary Information

Green polymer-based nanocomposites containing ceria and their use in the process of stem cell proliferation

Iosif V. Gofman[1,*], Alexandra L. Nikolaeva[1], Albert K. Khripunov[1], Elena M. Ivan'kova[1], Anton S. Shabunin[2], Alexander V. Yakimansky[1,3], Dmitriy P. Romanov[4], Anton L. Popov[5,6], Artem M. Ermakov[5], Sergey O. Solomevich[7], Pavel M. Bychkovsky[7], Alexander E. Baranchikov[6], Vladimir K. Ivanov[6]

[1] Institute of Macromolecular Compounds, Russian Academy of Sciences, 199004, Saint Petersburg, Russia; a.l.nikolaeva@imc.macro.ru (A.L.N.); ivelen@mail.ru (E.M.I.); yakimasky@yahoo.com (A.V.Y.)
[2] H.Turner National Medical Research Center for Children's Orthopedics and Trauma Surgery, 196603, Pushkin, Saint-Petersburg, Russia; anton-shab@yandex.ru (A.S.S.)
[3] Saint Petersburg State University, Institute of Chemistry, 198504, Peterhof, Saint-Petersburg, Russia
[4] Institute of Silicate Chemistry, Russian Academy of Sciences, 199034, Saint Petersburg, Russia; dprom@mail.ru (D.P.R.)
[5] Institute of Theoretical and Experimental Biophysics, 142290, Pushchino, Moscow region, Russia; antonpopovleonid@gmail.com (A.L.P.); ao_ermakovy@rambler.ru (A.M.E.)
[6] Kurnakov Institute of General and Inorganic Chemistry, Russian Academy of Sciences, 119991, Moscow, Russia; a.baranchikov@yandex.ru (A.E.B.); van@igic.ras.ru (V.K.I.)
[7] Research Institute for Physical and Chemical Problems of the Belarusian State University, 220030, Minsk, Republic of Belarus; sergeysolomevich@gmail.com (S.O.S.); bychkovsky@tut.by (P.M.B.)
*Correspondence: gofman@imc.macro.ru

Table S1. Selected gene groups for PCR-RT analysis.

Function	Description	GeneBank	Symbol	Forward 5'-3'	Rewerse 5'-3'
Glutathione Peroxidases (GPx)	Glutathione peroxidase 1	NM_000581	GPX1	CCTCCCCTTACAGTGCTTGTTC	GCACACATGGCGCAATTG
	Glutathione peroxidase 2 (gastrointestinal)	NM_002083	GPX2	CCGATCCCAAGCTCATCATT	TCTCAAAGTTCCAGGCCACAT
	Glutathione peroxidase 3 (plasma)	NM_002084	GPX3	CATCCCCTTCAAGCAGTATGCT	GCCCGTCAGGCCTCAGTAG
	Glutathione peroxidase 4 (phospholipid hydroperoxidase)	NM_002085	GPX4	CCGATACGCTGAGTGTGGTTT	GCTCCTGCTTCCCGAACTG
	Glutathione peroxidase 5 (epididymal androgen-related protein)	NM_001509	GPX5	TCACCACACTCTCTTCCTGCAT	AGAGTGGGAATTCTGGCAGTATG
	Glutathione S-transferase pi 1	NM_000852	GSTP1	CAGGAGGGCTCACTCAAAGC	GTGAGGTCTCCGTCCTGGAA
	Glutathione transferase zeta 1	NM_001513	GSTZ1	CCCAGAACGCCATCACTTG	TGCCCGCTGTGCTCTGT
Peroxiredoxins (TPx)	Peroxiredoxin 1	NM_002574	PRDX1	CTGGGACCCATGAACATTCC	AAGACCCCATAATCCTGAGCAA
	Peroxiredoxin 2	NM_005809	PRDX2	TCCTTCGCCAGATCACTGTTAA	CAGCCGCAGAGCCTCATC
	Peroxiredoxin 3	NM_006793	PRDX3	GCATTTGAGCGTCAACGATCT	TCACCAAGCGGAGGGTTTC
	Peroxiredoxin 4	NM_006406	PRDX4	GAGGCATCCCGGGTATCG	GGCTTGGAAATCTTCGCTTTG
	Peroxiredoxin 5	NM_181652	PRDX5	AGATGATTCGCTGGTGTCCAT	ACTATGCCATCCTGTACCACCAT
	Peroxiredoxin 6	NM_004905	PRDX6	GGCCGCATCCGTTTCC	CCCGAGGGTGGGAGAAGA
Other Peroxidases	Catalase	NM_001752	CAT	CAGGGCATCAAAAACCTTTCTG	CGGATGCCATAGTCAGGATCTT
	Cytochrome b-245, beta polypeptide	NM_000397	CYBB	CCTTTGAGTGGTTTGCAGATCTG	AGCCGGCATTGTTCCTTTC
	Cytoglobin	NM_134268	CYGB	GCAGCACCTCGAGCAGAAG	CCTTGGCACCCAGAAATGG
	Dual oxidase 1	NM_175940	DUOX1	TGAGCGGCACTTCCAGAAG	GACGGCCAAAGTGGGTGAT
	Dual oxidase 2	NM_014080	DUOX2	CCTTCGAGCCCTTCTTCAACT	CAGCTGAACACCCCGATCTT
	Lactoperoxidase	NM_006151	LPO	CAAGCTTTTCCAGCCAACTCA	CCGGCAACGCTGTGTGT
	Myeloperoxidase	NM_000250	MPO	CCTGAAATTGGCGAGGAAACT	GCCGCCCATCCAGATGT
	Prostaglandin-endoperoxide synthase 1	NM_000962	PTGS1	TGTTCGGTGTCCAGTTCCAATA	TGCCAGTGGTAGAGATGGTTGA
	Prostaglandin-endoperoxide synthase 2	NM_000963	PTGS2	AATTGCTGGCAGGGTTGCT	GGTCAATGGAAGCCTGTGATACTT
Other Antioxidants	Albumin	NM_000477	ALB	TGAGAAAACGCCAGTAAGTGACA	GAAAAGCATGGTCGCCTGTT
	Apolipoprotein E	NM_000041	APOE	CTGCGTTGCTGGTCACATTC	CTCTGTCTCCACCGCTTGCT
	Glutathione reductase	NM_000637	GSR	TGCAGGGACTTGGGTGTGA	GCCTTCGTTGCTCCCATCT
	Metallothionein 3	NM_005954	MT3	AGTGCGAGGGATGCAAATG	GCCTTTGCACACACAGTCCTT
	Sulfiredoxin 1	NM_080725	SRXN1	TGCTGTATCCCCAAGAATCATG	GCTAGTTTGGCCCTTCCTCTTC
	Superoxide dismutase 1, soluble	NM_000454	SOD1	TGGTGTGGCCGATGTGTCT	GTGCGGCCAATGATGCA
	Superoxide dismutase 2, mitochondrial	NM_000636	SOD2	TCCGCAGAAAGGAACATTAAGG	TGACCTCCATTCTTTGCTCTCA
	Superoxide dismutase 3, extracellular	NM_003102	SOD3	GCGGAGCCCAACTCTGACT	TGCCAGATCTCCGTGACCTT
Genes Involved in Reactive Oxygen Species (ROS) Metabolism	Arachidonate 12-lipoxygenase	NM_000697	ALOX12	CCACCCACCACCAAGGAA	TGCCGGACATCAGGTAGTGA
	Nitric oxide synthase 2, inducible	NM_000625	NOS2	CCGCATGACCTTGGTGTTT	TCCAGCATCTCCTCCTGGTAGA
	NADPH oxidase 4	NM_016931	NOX4	AAGAGCCCAGATTCCAAGCTAATT	CGGCACAGTACAGGCACAAA
	NADPH oxidase, EF-hand calcium binding domain 5	NM_024505	NOX5	AGGCACCAGAAAAGAAAGCATACT	ATGTTGTCTTGGACACCTTCGAT
	Uncoupling protein 2 (mitochondrial, proton carrier)	NM_003355	UCP2	CAGTTCTACACCAAGGGCTCTGA	CCTGTGGTGCTGCCTGCTA
	Aldehyde oxidase 1	NM_001159	AOX1	GGTGTTCCGTGTTTTTCGCTAT	GGTCCATGCAGGCCTCTCT

Function	Description	GeneBank	Symbol	Forward 5'-3'	Rewerse 5'-3'
	BCL2/adenovirus E1B 19kDa interacting protein 3	NM_004052	BNIP3	TCCATCTCTGCTGCTCTCTCATT	AGGTTGTCAGACGCCTTCCA
	Epoxide hydrolase 2, cytoplasmic	NM_001979	EPHX2	AACTGGGCCTCTCTCAAGCA	AGCCATGTACCACACCAGCAT
	MpV17 mitochondrial inner membrane protein	NM_002437	MPV17	TCTATGGCCTGCTGTGCAGTT	GGACAACGGCCAACCTGTA
	ATX1 antioxidant protein 1 homolog (yeast)	NM_004045	ATOX1	TGCTTGCAACCCTGAAGAAA	GGACCAGGCCCCTGCTA
	Chemokine (C-C motif) ligand 5	NM_002985	CCL5	TGCATCTGCCTCCCCATATT	AGTGGGCGGGCAATGTAG
	24-dehydrocholesterol reductase	NM_014762	DHCR24	CATGCTGGTGCCCATGAAG	GACGTGGATGTCGTTTTGGAA
	Forkhead box M1	NM_021953	FOXM1	AGGAAACGCTGCCCATCTC	CGTGAGCCTCCAGGATTCAG
	Ferritin, heavy polypeptide 1	NM_002032	FTH1	CTGGCTTGGCGGAATATCTCT	GCCCGAGGCTTAGCTTTCAT
	Glutamate-cysteine ligase, modifier subunit	NM_002061	GCLM	CCGCCTGCGGAAGAAGT	CATTCAAGGTTTTTGGATACAATCA
	Glutathione synthetase	NM_000178	GSS	GCAGGAAAAGACACTCGTGATG	CATGCTCGATGGCTTTGGT
	Heme oxygenase (decycling) 1	NM_002133	HMOX1	TCCGATGGGTCCTTACACTCA	GCCTGCATTCACATGGCATA
	Heat shock 70kDa protein 1A	NM_005345	HSPA1A	GCTGATTGGCCGCAAGTT	TGGAAAGGCCAGTGCTTCAT
	Mannose-binding lectin (protein C) 2, soluble	NM_000242	MBL2	AGTGAAGGCCTTGTGTGTCAAGT	TCCATTCTCTGCAGCATTCCT
	NAD(P)H dehydrogenase, quinone 1	NM_000903	NQO1	CAGCAGACGCCCGAATTC	TGGTGTCTCATCCCAAATATTCTC
	Ring finger protein 7	NM_014245	RNF7	AAAGGAAAGAGCTCCAAATTGAATC	CATAAGCATGCAAAAAGTTCTCTGA
	Sirtuin 2	NM_012237	SIRT2	GCTGGAACAGGAGGACTTGGT	TGGCGCTGACGCAGTGT
	Sequestosome 1	NM_003900	SQSTM1	GGAAGGTGAAACACGGACACTT	ACGTGGGCTCCAGTTTCCT
Pathway Activity Signature Genes	Aldo-keto reductase family 1	NM_001354	AKR1C2	GATTGCCCTGCGCTACCA	TGTCTGATGCGCTGCTCATT
	BCL2-associated athanogene 2	NM_004282	BAG2	CTCACCGTTGAAGTGTCAGTAGAAA	ATCAATAATCCTTGTGGCATGCT
	Four and a half LIM domains 2	NM_001450	FHL2	CCTGCAGGAAGCAGCTGTCT	AGTTCAGGCAGTAGGCAAAGTCA
	Galactosidase, alpha	NM_000169	GLA	GGATGGCTCCCCAAAGAGAT	GGCGAATCCCATGAGGAAA
	Heat shock protein 90kDa alpha (cytosolic), class A member 1	NM_001017963	HSP90AA1	TTGGCAGTGAAGCATTTTTCAG	GAGCACGTCGTGGGACAAA
	Phospholysine phosphohistidine inorganic pyrophosphate phosphatase	NM_022126	LHPP	TGCGCACCGGGAAGTT	CACGTACCCATCAGCCTTCA
	Trafficking protein particle complex 6A	NM_024108	TRAPPC6A	GGTGTTCCAGAAGCAGATGGA	AGCTGTTGTCTTGCAGGACGTA
Mitochondrial dysfunction	Mitochondrial ribosomal protein L43	NM_176794	MRPL43	CAGTTGCACCGCAGATCCT	GGAAGATCGGATGACTGAACTGA
	NADH dehydrogenase (ubiquinone) 1 beta subcomplex, 11, 17.3kDa	NM_019056	NDUFB11	GCAGCACCTTTGTGGCCTAT	TCCCATCCCACGCTCTTG
	Polymerase (RNA) mitochondrial (DNA directed)	NM_005035	POLRMT	CACAGGTGCTGGAAGGTTTCA	CCGTACACCACCGTCATCAC
	Sirtuin 1	NM_012238	SIRT1	TGAGCCTGATGTTCCAGAGAGA	AGCTTCATTAATTGCCTCTTGATCAT
	Sirtuin 3	NM_012239	SIRT3	CCAGTGGCATTCCAGACTTCA	GATCGTACTGCTGGAGGTTGCT
	Transcription factor B1, mitochondrial	NM_016020	TFB1M	GCCATCGAGGGCTCAGAA	CAGCCTGCCCGTGCTTT
	Transcription factor B2, mitochondrial	NM_022366	TFB2M	AAGGCGTCTAAGGCCAGCTT	TTTGCGCCAGGGTCTCA
	Copper chaperone for superoxide dismutase	NM_005125	CCS	GCCGCGCCATCTTCAG	ATCAGGCTGCGGCCAAT
	Selenoprotein P, plasma, 1	NM_203472	SELENOS	CTGAAACGGAAATCGGACAGA	CGCCTCCTTCACCAGACAAC
Anti Apoptotic	B-cell CLL/lymphoma 2	NM_000633.2	BCL2	CTGGGATGCCTTTGTGGAACT	AGACAGCCAGGAGAAATCAAACAG
	aculoviral IAP repeat containing 3	NM_001165.4	BIRC3	GGACAGGAGTTCATCCGTCAAG	TCTCCTGGGCTGTCTGATGTG
	myeloid cell leukemia 1	NM_021960.4	MCL1	CACGAGACGGCCTTCCAA	CACTCGAGACAACGATTTCACATC

Function	Description	GeneBank	Symbol	Forward 5'-3'	Rewerse 5'-3'
	TNF receptor-associated factor 2	NM_021138.3	TRAF2	GGCCGTCTGTCCCAGTGAT	TTCGTGGCAGCTCTCGTATTC
Autophagy	autophagy related 3	NM_022488.4	ATG3	CCATTGAAAATCACCCTCATCTG	CACCTCAGCATGCCTGCAT
	autophagy related 12	NM_004707.3	ATG12	CCCGGGAACAGAGGAACCT	GGAGTGTCTCCCACAGCCTTT
	nuclear factor of kappa light polypeptide gene enhancer in B-cells 1	NM_003998.3	NFKB1	GGCTACACCGAAGCAATTGAAG	CAGCGAGTGGGCCTGAGA
	ribosomal protein S6 kinase, 70kDa, polypeptide 1	NM_003161.3	RPS6KB1	TGGCATAGAGCAGATGGATGTG	AGAGTTCGGCTGTCGTATTGGA
Necrosis	coiled-coil domain containing 103	NM_213607.2	CCDC103	GCTGCAAGGGCTTGTTTCAG	GCCCCTCCTTCACGGATCT
	forkhead box I1	NM_012188.4	FOXI1	CGCCTCACTCTCAGCCAGAT	CCGGCCTTGCTCTTGTTGTA
	junctophilin 3	NM_020655.3	JPH3	CCAGGATCACTGCCAAAGAGTT	CGCTTCGGCCTCTGGTACT
	RAB25, member RAS oncogene family	NM_020387.2	RAB25	TGTCTTCAAGGTGGTGCTGATC	CGCGTGAATCGGGAGAGTAG
Pro apoptotic	BCL2-associated X protein	NM_004324.3	BAX	GTGGCAGCTGACATGTTTTCTG	GCAAAGTAGAAAAGGGCGACAA
	CD40 molecule, TNF receptor superfamily member 5	NM_001250.4	CD40	ACACTGCCACCAGCACAAATACT	CTGTTTCTGAGGTGCCCTTCTG
	CASP8 and FADD-like apoptosis regulator	NM_003879.5	CFLAR	GTGTGTATGGTGTGGATCAGACTCA	GGCATGAATCTCCCATGAACA
	Fas cell surface death receptor	NM_000043.4	FAS	GAATCATCAAGGAATGCACACTCA	AAAGCCACCCCAAGTTAGATCTG
	Tumor necrosis factor receptor superfamily, member 10a	NM_003844.3	TNFRSF1	CTGGCGCTTGGGTCTCCTA	TGCGTTGCTCAGAATCTCGTT
House keeping	glyceraldehyde-3-phosphate dehydrogenase	NM_002046.5	GAPDH	GTGGAAGGACTCATGACCACAGT	GCCATCACGCCACAGTTTC
	ribosomal protein, large, P0	NM_001002.3	RPLP0	ATGCAGCAGATCCGCATGT	TTGCGCATCATGGTGTTCTT
	beta-actin	XM_006715764.1	Actin	TCGTGCGTGACATTAAGGAGAA	AGCAGCCGTGGCCATCT
